# The Effects of Acupuncture Combined with Auricular Acupressure in the Treatment of Chloasma

**DOI:** 10.1155/2018/6438458

**Published:** 2018-04-22

**Authors:** Xing Wu, Yu Xiang

**Affiliations:** ^1^Clinical Medical College of Acupuncture, Moxibustion and Rehabilitation, Guangzhou University of Chinese Medicine, Guangzhou, Guangdong 510006, China; ^2^School of Medical Information Engineering, Guangdong Pharmaceutical University, Guangzhou, Guangdong 510006, China

## Abstract

**Objective:**

To investigate the effectiveness of acupuncture combined with auricular acupressure in chloasma treatment.

**Methods:**

A prospective, randomized controlled assessor-blind clinical trial was performed and 135 patients were assigned into acupuncture combined with auricular acupressure (A), acupuncture (B), and control (C) groups, each with 45 patients. For groups A and B, body and facial acupuncture were applied for 2 months. For group A, auricular acupressure was applied concomitantly. For group C, vitamins C and E were prescribed for 3 months. Primary outcome measure was the therapeutic effects while secondary outcome measure was safety evaluation.

**Results:**

The total effective rate was 95.6%, 91.1%, and 75.6% for groups A, B, and C (*P* < 0.01 between groups A and C; *P* < 0.05 between groups B and C). The posttreatment estradiol (E_2_) levels in groups A and B were significantly decreased while the progesterone (P_4_) levels were significantly increased compared to pretreatment (*P* < 0.01 and *P* < 0.05, resp.). The differences were significant compared to group C (*P* < 0.01 and *P* < 0.05, resp.). No adverse events occurred.

**Conclusion:**

Acupuncture combined with auricular acupressure could significantly increase the therapeutic effect of chloasma treatment and could be better than vitamins C and E.

## 1. Introduction

Chloasma (or melasma) is a skin pigmentation disease characterized by symmetrical distribution of brown or dark brown patches on the face zygomatic region, forehead, nose, or mouth, typically with irregular sizes and defined margin [1]. Although it is not a severe disorder, location on the face makes it a significant cosmetic condition and imposes emotional impact to the individual, affecting quality of life [2–4].

Chloasma is seen primarily in women. It is thought to be caused by increased stimulation of melanocytes secondary to estrogen and progesterone, ultraviolet light, thyroid dysfunction, and genetic predisposition [5–8]. Chloasma lesions have been found to be associated with increased expression of vascular endothelial growth factor (VEGF) [9–11].

Topical hydroquinone and tretinoin have been used commonly for treatment. Combined therapy such as topical hydroquinone, tretinoin, and corticosteroids has been thought to increase the efficacy of monotherapy. Flavonoid extracts and other compounds have been investigated for their hypopigmentation effects; however, their efficacy and safety indicate more study is needed before they can be recommended for use. Other modalities include chemical peels and laser treatment [12].

Use of acupuncture in skin disease treatment has been a long history and belongs to the category of external treatment in Traditional Chinese Medicine (TCM). It is safe and simple and has good efficacy. Acupuncture can improve the balance of yin and yang, smooth the flow of blood [13], regulate body's immune and endocrine function, and enhance and improve body's resistance [14]. Findings from previous studies suggest that acupuncture can alter the pathogenic pathway which cause hyperpigmentation by decreasing the level of oestrogen, lipid peroxidation, and *α*-melanocyte-stimulating hormone and increasing the level of superoxide dismutase [15].

Acupuncture has been recognized by the World Health Organization as a promising therapeutic modality in chloasma [16]. It has been found to be beneficial and safe and has been widely accepted in China with no known related adverse events. Previous systematic review found that acupuncture improves outcome measures in the treatment of multiple dermatologic conditions including chloasma [17]. However, relevant studies in the treatment of chloasma are still relatively scarce [18].

Auricular acupressure (also known as “auriculotherapy” does not require the use of needle and therefore is noninvasive. Previous studies have shown that auricular acupressure alone or in combination with acupuncture or herbal drugs is an effective method for chloasma treatment [19]. It has great advantage in treating chloasma with its unique method. Through harmonizing the internal zang-fu and yin-yang and smoothing the qi and blood, its effectiveness in treatment is achieved both internally and externally. However, its mechanism is uncertain and there is still lack of randomized clinical trials [19]. Its combination with acupuncture may enhance the therapeutic effect in chloasma treatment.

Vitamin C is a natural antioxidant [20] and has been used as a lightening agent in oral and topical forms [21, 22]. Its mechanisms of action in the treatment of chloasma include interacting with the copper ions at the tyrosinase active site [23] and inhibiting melanogenesis by acting as a reducing agent in various oxidative steps of melanin formation [20]. Vitamin E is the major lipophilic antioxidant in humans and can be effective in the treatment of chloasma through various mechanisms. These include photoprotective activity, interfering with lipid peroxidation of the melanocyte membranes, increasing intracellular glutathione content [23], inhibiting tyrosinase [20], and inhibiting tyrosinase hydroxylase activity [21].

Thus, in this study we compare the effectiveness and safety of acupuncture combined with auricular acupressure, acupuncture, and conventional treatment with oral vitamin C and vitamin E. We hypothesized that the addition of auricular acupressure will improve the therapeutic effect of chloasma treatment.

## 2. Materials and Methods

### 2.1. Ethical Approval

This study was approved by the Ethics Committee of the Guangzhou University of Chinese Medicine and meets the standards of the Declaration of Helsinki in its revised version of 1975 and its latest amendments of 1996 [24]. Written informed consent was obtained from all participants prior to their inclusion in the study. Study is reported in compliance with the CONSORT statement (http://www.consort-statement.org) [25] as well as STRICTA (Standards for Reporting Interventions in Clinical Trials of Acupuncture) [26].

### 2.2. Clinical Data

A randomized controlled clinical trial was carried out on the female outpatients of the authors hospital from January 2016 to December 2016. Female chloasma patients were invited to participate in the study.

*Inclusion Criteria*. They included all female patients diagnosed with chloasma (age 18 to 60 years) and 1–30 years in course of chloasma who were willing to participate.

*Exclusion Criteria*. They included (1) patients with systemic disease or severe skin disease; (2) patients using light sensitive drugs at present; (3) patients treating the disease with fruit acid treatment within one month; (4) patients with bleeding tendency or receiving anticoagulants or antiplatelet aggregation drugs; (5) patients with psychiatric problems or other neurological disabilities; (6) pregnant or breastfeeding women; (7) patients suffering from other pigmentary disorders.

The purpose and procedures of the study were explained to eligible patients and their families and personal particulars such as age, gender, occupation, marital status, duration of chloasma, family history of chloasma, use of hormonal therapy and cosmetics, sun exposure, and history of smoking were recorded. The primary database was the data collected by the assessors before and after treatment.

### 2.3. Follow-Up

Follow-up refers to continuing intervention after first visit. A card appointment was made for each patient which consists of the schedule of appointment. They were reminded to keep it in a safe place and bring it each visit. The card was ticked at the column next to the date of appointment at the end of each visit. They were also reminded to turn up for their next visit. Telephone calls were also arranged to remind patients who did not turn up in their visits.

#### 2.3.1. Standard of Rejection

Patients were removed from the study if they (1) violated the therapeutic plan in manipulation; (2) suspended two treatments in the therapeutic period; (3) used other methods to help relief chloasma symptoms.

#### 2.3.2. Standard of Drop-Out

Patients were considered drop-outs of the study if they (1) failed to visit; (2) willingly quit the therapeutic process or could not persist in treatment due to unexpected conditions; (3) had poor obedience with incomplete data during the clinical trial.

#### 2.3.3. Standard of Suspension

Patients were suspended if they: (1) could not tolerate the treatment or had adverse reactions during treatment; (2) refused to be treated or continued to use drugs or other treatments during the therapeutic period.

### 2.4. Study Design and Subjects

This is a single-center, prospective, randomized controlled assessor-blind clinical trial. After completing the informed consent, a total of 135 patients were finally selected and randomly assigned into 3 groups: acupuncture combined with auricular acupressure (Group A, *n* = 45), acupuncture (Group B, *n* = 45) and control with Vitamin C 200 mg and vitamin E 100 mg (Group C, *n* = 45).

### 2.5. Diagnostic Criteria

Patients were diagnosed based on the standards for Clinical diagnosis and efficacy criteria for Chloasma established by the Professional Board of Dermatology and Venereology of China Association of Combined Chinese and Western Medicine [27]. The criteria for diagnosis were: (1) light or dark brown plaques with clear margin occurring in the face and symmetrically distributed with no inflammatory manifestation and scale (2) no obvious subjective symptoms (3) mostly occurs in postpuberty female (4) severe in summer and alleviated in winter (5) exclusion from endocrine diseases or other diseases that can induce pigmentation (6) over 20% increase of the average optical density in the area with pigmentation as compared to the average optical density of the patient's face.

### 2.6. Randomization and Blinding

Randomization of subjects of study was performed using computer generated random number tables. Allocation concealment was achieved using sequentially numbered, opaque, sealed envelopes by a researcher not to have any contact with patients. The assessors (practitioners) who did the scoring for pre- and post-treatment and therapeutic effects evaluation were blinded to the type of intervention each patient received. The acupuncture practitioner did not provide any clues regarding group assignments to the assessors. The results were averaged from two assessors.

### 2.7. Treatment Groups (Group A and Group B)

Acupuncture points in the body region included Quchi (LI 11), Hegu (LI 4), Xuehai (SP 10), Zusanli (ST 36) and Sanyingjiao (SP 6). Taichong (LR 3) and Ligou (LV 5) were added for stagnation of Liver qi; Yinlingquan (SP 9) and Fenglong (ST 40) were added for Spleen deficiency and dampness; Shenshu (BL 23) and Taixi (KI 3) were added for Kidney deficiency. Acupoints were applied bilaterally. After local sterilization with 75% alcohol, disposable filliform needles size 0.25 × 25 mm (Hwato brand, Suzhou Medical Appliances, Suzhou, Jiangsu, P.R. China) were inserted beneath the skin of these points. Reinforcing-reducing method was performed at each point. Upon arrival of qi, needles were inserted to a depth of 5–10 mm and retained for 30 minutes. Intervention was performed once every 10 min interval, for a total of 4 times per treatment. Needle-flicking was applied for each point 1-2 times before withdrawal of needle. Treatment was given once every other day. Fifteen treatments constituted a therapeutic course, for a total of 2 courses.

In addition, facial acupuncture was performed by inserting acupuncture needles at the rims of the chloasma lesion. Transverse penetrating method was performed. After local sterilization with 75% alcohol, disposable facial needles size 0.18 × 13 mm (Hwato brand, Suzhou Medical Appliances, Suzhou, Jiangsu, P.R. China) were used. With the needle tip and skin surface forming an angle of 15°, needle were inserted to a depth of 0.2 mm along the margins of the chloasma toward the center of the affected area. Total needle used ranged from 5–10 depending on the size of chloasma. Needles were retained for 30 min. Treatment was applied once every other day. Fifteen treatments constituted a therapeutic course, for a total of 2 courses.

Traditional Chinese needle acupuncture was used in all of the above cases. Therapeutic effects were evaluated after two consecutive courses of the above treatments.

For acupuncture combined with auricular acupressure (Group A), auricular acupressure was applied concomitantly. The auricular points included Shenmen (MA-TF 1), Lung (MA-IC 1), Endocrine (MA-IC 3), Subcortex (MA-AT 1), and Cheek (MA-LO 7). After local sterilization with 75% alcohol, the auricular sensitive point was detected with auricular point probe (Zhongyan Taihe, Beijing Jianlekang Medical Instrument Co., Ltd., Beijing, P.R. China) to determine the treatment points. Vaccaria seeds (Dingyao, Jiangmen Xinli Medical Apparatus and Instruments Co., Ltd. Guangdong, P.R. China) were fixed with 0.5 × 0.5 cm adhesive tapes at the selected auricular points. The acupoints were pressed immediately for 2-3 min until it produces the sensation of soreness, fullness or numbness (deqi). The patients were guided and advised to press the seeds 3-4 times a day by herself. The adhesive tapes were replaced once every other weeks, and the acupoints from the two ears were treated alternately. 15 sessions constituted one course of treatment, for a total of 4 courses.

For the above treatments, all patients was treated by a licensed TCM practitioner who had more than 15 years of experience. Each subject was treated by the same practitioner for each session. For auricular acupressure, during follow-up visits the practitioner would enquire and ensure that the patients were performing it correctly, such as making them to explain how they did it, checking whether the correct acupoints were applied and that they felt the “deqi” sensation.

### 2.8. Control Group (Group C)

Patients in the control group were given oral medication of Vitamin C 200 mg and Vitamin E 100 mg, 3 times daily. The therapeutic effect was evaluated after a 3-months treatment. No acupuncture or sham acupuncture was performed on the control group.

### 2.9. Treatment Duration

The total duration of treatment for group A and group B were 2 months while for control group C, vitamin C and E were given for 3 months. Based on our previous experience and literature, this duration is applicable to patients to achieve the optimal effects of treatment.

### 2.10. General Precautions

During the treatment period, patients in all groups were advised to withdraw other medications, avoid prolonged exposure to sunlight and prohibited to use cosmetics and eat oily, raw, cold or spicy food. All participants was observed for any adverse events at each visit.

### 2.11. Criteria for Therapeutic Effect [27]

The area and color of the skin lesion combined with the clinical condition were taken as factors for evaluation of the therapeutic effect.

  Area of the skin lesion: Score 0: no skin lesion; 1: area < 1 cm^2^; 2: 1–3 cm^2^; 3: > 3 cm^2^
  Color of the skin lesion: Score 0: normal color; 1: light brown; 2: brown; 3: dark brown
  The total score was the sum of the two. Index reduction after treatment = (total score before treatment − total score after treatment)/total score before treatment.
  Basically cured: reduction of the area by >90% by naked eye vision, color of the chloasma basically disappear and index reduction after treatment ≥ 0.8.
  Markedly effective: reduction of area by >60% and ≤90% by naked eye vision, color significantly getting lighter and index reduction after treatment ≥ 0.5 and <0.8.
  Effective: reduction of area by >30% and ≤60% by naked eye vision, color getting lighter and index reduction after treatment ≥ 0.3 and <0.5.
  Ineffective: reduction of area by ≤30% by naked eye vision, color not getting lighter and index reduction after treatment < 0.3.
  Total effective rate for each group (%) = (Basically cured + markedly effective + effective)/(number of patients in each group) × 100%
  The assessment of therapeutic effects were performed after completed intervention.

### 2.12. Safety Evaluation

Any adverse events such as bruising, needle site bleeding, and increased pain [28, 29] would be recorded and intervention would be stopped immediately.

### 2.13. Test Indicators

10 ml of venous blood sample was drawn from all patients after overnight fasting at the 3rd–5th day of menstrual cycle in the mid follicular phase for pre- and post-treatment assessment of follicle stimulating hormone (FSH), luteinizing hormone (LH), estradiol (E_2_) and progesterone (P_4_) by chemiluminescence method. All blood samples were taken at 8–10 am in the same designated clinic.

### 2.14. Statistical Analysis

Data were presented as mean ± standard deviation for continuous variables or as percentages for categorical variables. To test the effectiveness of acupuncture treatment, a nonparametric ANOVA (Kruskal-Wallis *H* test) followed by pairwise multiple comparisons was used to compare the pre-post differences of the same group. For comparisons with control group, Wilcoxon signed-rank test was used. Statistical analysis was performed using SPSS software (version 19.0, SPSS, Chicago, Illinois, USA). *P* < 0.05 was considered as statistically significant.

The analyses were performed on an intention-to-treat basis, with missing data replaced by the principle of last observation carried forward.

### 2.15. Sample Size Calculation

The number of patients was calculated based on the outcome of the previous study [30] and the period of study. With a 5% chance of type 1 error (*α* = 0.05) and a possible 10% follow-up loss, we approximate 45 initial participants in each group would be required to have a sufficient sample size.

### 2.16. Outcome Measures

(1) to compare the effectiveness of acupuncture combined with auricular acupressure, acupuncture and conventional treatment with oral vitamin C plus vitamin E.
(2) to observe for any adverse events occurred.

## 3. Results

**Figure 1:**
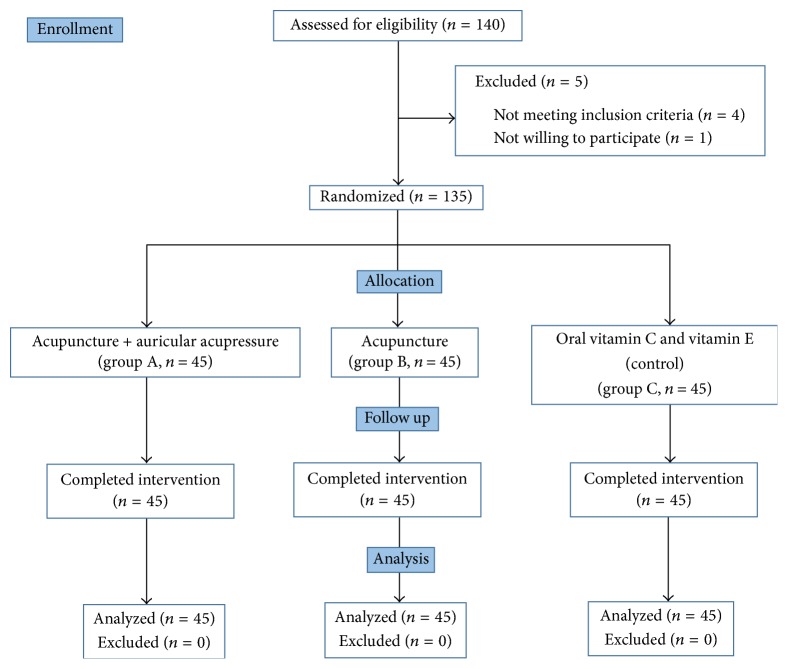
Flow chart of study.* Note*. *n*: number of patients.

A total of 135 patients met the inclusion criteria and was recruited in the study. There was no drop-outs from the study. Flow chart of study was as shown in Figure 1.

### 3.1. Baseline Characteristics

Participants in the both treatment groups were similar in terms of age, weight, height, BMI, duration of chloasma, and history in a first-degree relative. The mean age of presentation was 37.5 ± 5.2, 38.1 ± 4.8, and 36.9 ± 5.0 for groups A, B, and C, respectively. The mean duration of chloasma was 5.5 ± 1.2, 6.0 ± 1.0, and 5.7 ± 1.3 for groups A, B, and C, respectively. The baseline characteristics of the patients were summarized in Table 1.

### 3.2. Evaluation of Therapeutic Effect

Comparison of the treatment outcome was summarized in Table 2. The total effective rate was 95.6%, 91.1%, and 75.6% for group A, B, and C, respectively, with a significant difference of *P* < 0.01 for comparison between groups A and C and *P* < 0.05 for comparison between groups B and C, indicating that acupuncture + auricular acupressure and acupuncture were superior to control group while the effectiveness of acupuncture + auricular acupressure was the most significant compared to control.

### 3.3. Hormone Levels for Pre- and Posttreatment

The levels of FSH and LH of patients for pre- and posttreatment in groups A, B, and C were not significantly different. The levels of E_2_ for posttreatment in group A and group B were significantly decreased (*P* < 0.01 and *P* < 0.05, resp.) while the levels of P_4_ were significantly increased (*P* < 0.01 and *P* < 0.05, resp.) compared to the pretreatment level of the same group. The decrement of E_2_ in groups A and B was significantly different from the control group (group C) (*P* < 0.01 and *P* < 0.05, resp.) while the increment of P_4_ in groups A and B was also significantly different from the control group (group C) (*P* < 0.01 and *P* < 0.05, resp.) The levels of decrement and increment of E_2_ and P_4_ between groups A and group B were not significantly different (Table 3).

### 3.4. Adverse Events Reporting

No adverse events occurred throughout the procedures and during follow-ups.

## 4. Discussion

Chloasma is a hyperpigmented disease occurring in the facial area. TCM believes that the pathogenesis of chloasma is related to dysfunction of the zang-fu organs, derangement of qi and blood, obstruction of channels and collaterals, stagnation of qi and blood stasis, and malnutrition [31, 32]. The commonly seen chloasma type is Liver-qi stagnation, Spleen deficiency, Kidney-yin-deficiency, and Kidney-yang-insufficiency [33].

The pathophysiology of chloasma is uncertain; however, many causative factors have been implicated and these include exposure to UV radiation and visible light [34], genetic influences, pregnancy oral contraceptives and hormone replacement therapy, ovarian tumors, and cosmetics [35]. The most important factor in the development of chloasma is exposure to sunlight. Ultraviolet radiation can cause peroxidation of lipids in cellular membranes, leading to generation of free radicals, which could stimulate melanocytes to produce excess melanin. Sunscreens that primarily block UV-B radiation (290–320 nm) are unsatisfactory because longer wavelengths (UV-A and visible radiation, 320–700 nm) also stimulate melanocytes to produce melanin [36]. The principles of therapy in chloasma are to provide protection from ultraviolet (UV) light, retard the proliferation of melanocytes, inhibit the formation of melanin and melanosomes, and promote the degradation of melanin pigments by keratinocytes or melanophages [37, 38]. In this study, 57 (42.2%) of patients have previous history of sun exposure, 2 (1.5%) of patients have previous history of hormonal therapy, 2 (1.5%) of patients have previous history of cosmetic use, and 60 (44.4%) of patients have a history of chloasma in their first-degree relative.

There were no drop-outs from the study probably because besides the steps taken to reduce the possibility of drop-outs, the patients mostly came from the same province and were staying nearby. They are very much concerned and keen on having a curative effect for their disease, especially due to cosmetic reason particularly for the professionals and also housewives. Furthermore, no adverse events occurred. Before participation, we had also explained to them the importance of compliance to treatment and, during follow-up, they were reminded to turn up for their next visits.

Vitamin C converts deep, oxidized pigments to light, reduces pigments, prevents oxidation of melanin catabolism, and inhibits the formation of melanin [37, 39]. Vitamin E has been shown to cause depigmentation by interference with the lipid peroxidation of melanocyte membranes, increase in intracellular glutathione content, and inhibition of tyrosinase [40]. Vitamin E alone has shown minimal efficacy in the treatment of chloasma [39]. Combined preparation of vitamins C and E has been shown to produce better clinical improvement in chloasma treatment than vitamin C alone [39]. High doses of vitamin C can cause occasional abdominal pain and diarrhea, resulting from the osmotic effects of unabsorbed quantities of vitamin C [41]. As a safety guidance, tolerable upper intake levels have been established by the Food and Nutrition Board, Institute of Medicine, at 1000 mg for vitamin E and 2000 mg for vitamin C in adults [41]. A clinical trial concluded that vitamin E supplements of </=1600 IU (1073 mg RRR-alpha-tocopherol or the molar equivalent of its esters) and vitamin C supplements of </=2000 mg/d are safe for most adults [41]. In this study, combined treatment of oral vitamins C and E used in control group was within the safe therapeutic range. Oral vitamins C and E were selected as control in this study as they are noninvasive, are commonly used, have almost no side effects if used within the therapeutic range, and thus produce less psychological effects such as fear and anxiousness to patients. It can also easily be found in natural remedies.

Acupuncture activates blood circulation, promotes metabolism of epidermal cells, and balances zang-fu to regulate various system. Acupuncture at the facial point and the corresponding damaged skin areas can also promote regeneration of cells and extinction of pigmented spots [32].

Quchi (LI 11) and Hegu (LI 4) are located at the Large Intestine Meridian of Hand Yangming. Quchi is the He-Sea point of the Large Intestine. It can regulate qi and blood and activate meridian. Hegu (LI 4) is an important point for treating diseases of the mouth and face. It has the effect of regulating qi and blood. Xuehai (SP 10) and Sanyinjiao (SP 6) are located at the Spleen Meridian of Foot Taiyin. Xuehai can activate qi flow and promote circulation of blood to remove blood stasis. Sanyinjiao strengthens the Spleen, stimulates the function of the Liver, smooths the flow of Liver qi, tonifies Kidney, nourishes the blood and yin, moves the blood, eliminates stasis, and cools the blood. Zusanli (ST 36) is located at the Stomach Meridian of Foot Yangming. It regulates and tonifies Spleen and Stomach and strengthens vital qi. Zusanli when combined with Sanyinjiao can enhance the function of strengthening the Spleen and regulating qi flow. This pacifies the Liver, strengthens the Spleen, and regulates qi and blood circulation. Previous systematic review found that Xuehai (SP 10), Zusanli (ST 36), and Sanyingjiao (SP 6) were the body acupoints most often used in chloasma treatment [18].

Taichong (LR 3) is located at the Liver Meridian of Foot Jueyin. It is the Shu-Stream and Yuan-Source point of the Liver and is the main point for calming the Liver. It clears away damp heat from the Liver Meridian and tonifies Liver. Hegu when combined with Taichong can regulate Liver qi and balance yin and yang. Ligou (LV 5) is the Luo-Connecting point of the Liver Meridian of Foot Jueyin. It regulates Liver qi and removes damp heat of the Liver. Clinically, it is often combined with Yuan-Source points for the treatment of diseases.

Yinlingquan (SP 9) is located at the Spleen Meridian of Foot Taiyin. It regulates the Spleen and resolves dampness. Fenglong (ST 40) is located at the Stomach Meridian of Foot Yangming. It is the Luo-Connecting point of the Stomach Meridian. It calms the shen and activates the meridian. It is often combined with Yuan-Source points in the treatment of diseases.

Shenshu (BL 23) is located on the Bladder Meridian of Foot Taiyang. It strengthens the Kidney and is able to grasp qi. It is the most effective point to tonify the Kidney. Taixi (KI 3) is located at the Kidney Meridian of Foot Shaoyin. It is one of the most important acupoints for strengthening and restoring vital energy of the Kidney. The Kidneys are the root of all the Yin and Yang energy of the whole body, and this point can help to strengthen both yin and yang and can be used for Yin deficiency (feelings of heat, flushing, night sweats, and irritability) or Yang deficiency (feelings of cold, low energy, little motivation, poor digestion, and depression).

In the present study, surrounding needling therapy was used for facial acupuncture. Previous studies showed that surrounding needling therapy plus body acupuncture can produce good therapeutic effect in treating chloasma [30]. It can improve local circulation, promote metabolism of epidermis cell, diminish spots, and enhance muscle elasticity. Combining body acupuncture with syndrome differentiation, it regulates blood and qi of the whole body, regulates endocrine, promotes systemic blood circulation to achieve its therapeutic effect, and reduces relapses [30].

Auricular points are added to enhance the therapeutic effect. Since every organ of the body is represented upon the external ear, auricular acupuncture is considered a potential source for alleviating any disease [42]. Although stimulation of ear reflex points and body acupoints seems to be equally effective, auricular acupuncture tends to work more quickly than body acupuncture [42]. Body acupuncture and auricular acupuncture are often used with each other in the same session, or each procedure can be effectively applied separately [42]. Body and auricular acupuncture points can both be stimulated with the use of acupuncture needles, acupressure massage, or electroacupuncture. Compared to the other two, acupressure is easier to perform, less painful, and produces less nervousness to patients. For some patients who fear of needles, acupressure is a better choice for them and produces less psychological effects. The application is safe and easy to learn and practice and has less adverse events. Even the patients can be trained to perform it correctly on themselves. It saves time, is more convenient, and helps to reduce the frequency of visits and costs.

Shenmen (MA-TF 1) is the foremost point in the treatment of virtually every disease. Auricular Shenmen adds “dampness” to the body. It calms the spirit, tranquilizes the mind, and facilitates a state of harmony and serenity. It alleviates stress, pain, tension, anxiety, depression, insomnia, restlessness, and excessive sensitivity. The Lung point (MA-IC 1) also has a zang-fu connection. It contributes to one's energy level, as the Lungs are the master of qi. It is very applicable in skin problems and healing of mucous membranes, as the Lungs dominate the skin and mucous membranes. The Endocrine point (MA-IC 3) regulates all endocrine glands needed for homeostasis of the internal bodily environment and is sometimes referred to as the internal secretion point. Subcortex (MA-AT 1) regulates excitement and inhibition of the cerebral cortex. Proper utilization can greatly increase the therapeutic effect of auricular acupuncture. The Cheek point (MA-LO 7) is often used to relieve facial paresthesia, spasms, and acne.

The present study showed that acupuncture combined with auricular acupressure can produce better treatment results than acupuncture or control group. Combination of auricular acupressure may enhance the therapeutic effect of acupuncture. The ear and the meridian zang-fu are closely related. In the physiological aspect, the auricular acupoints are reaction points of qi and blood of the zang-fu. They adjust the balance of zang-fu yin and yang and smooth the meridians [43].

Modern medicine found that patients with chloasma tend to have hemodynamic abnormality, which corresponds to the TCM term of blood stasis [44]. It has been proved that chloasma has certain relationship with the increase of blood viscosity and the microcirculatory disturbances [45]. Acupuncture and auricular acupressure treat the underlying condition and affect deeper physiological changes by facilitating the natural self-regulation homeostatic mechanisms of the body [42]. Stimulating an acupoint can diminish overactive bodily functions or activate physiological processes that were deficient [42].

Along with other etiologic factors such as sun exposure and pregnancy drugs, hormones such as estradiol and progesterone have been found to be the major contributing factors in the development of chloasma [46]. The occurrence of chloasma with estrogen- and progesterone-containing oral contraceptive pills has been reported [36]. Chloasma has been shown to be associated with increased estradiol and decreased progesterone [47]. The results of this study showed that acupuncture combined with auricular acupressure or acupuncture alone could significantly decrease the level of pretreatment E_2_ and increase the level of P_4_. The difference of E_2_ and P_4_ levels in the pre- and posttreatment was also significant compared to the control group, with the results of the acupuncture combined with auricular acupressure group being the most significant. Acupuncture combined with auricular acupressure or acupuncture alone could regulate the level of E_2_ and P_4_. A previous study done by Mao et al. which compared the efficacy difference between the meridian cosmetology (with acupuncture and local surrounding needling therapy) and western medicine (with oral vitamin C and E for 3 months) for chloasma showed that the effective rate of the meridian cosmetology group was more superior to western medicine (92.6% versus 67.0%) with decreased level of estradiol (*P* < 0.01) and increased progesterone (*P* < 0.05) [47].

Previous systematic review and meta-analysis on acupuncture for chloasma showed favorable effects of acupuncture [48]. Its application is beneficial and safe [18, 48]. The encircling needling method was among the widely used in chloasma treatment [18]. Another systematic review on acupuncture as a treatment modality in dermatology [17] showed statistically significant greater clearance of chloasma in patients treated with acupuncture plus herbal medicine compared to those treated with oral vitamins C and E [32] while another study found that acupuncture could significantly lessen the size and lighten the color of chloasma [49]. A study on chloasma treated with auricular pressing and pricking found total effective rate of 87% in patients [50]. Previous study on auricular acupressure with* Vaccaria* seeds combined with body acupuncture in chloasma treatment showed an effective rate of 88.1% [51]. In this study, the application of auricular acupressure in addition to body and facial acupuncture showed increase in the effective rate in chloasma therapy. Auricular acupressure is economical and convenient. It is safe and not painful [43]. Insertion of auricular acupuncture is more simple to apply with little danger of damaging a critical blood vessel [42]. Application of auricular acupressure at the similar acupoints may produce similar effect and less invasive.

The limitations in this study were that therapeutic effect was examined under naked eye vision which might be subjected to bias of judgement. Patients with auricular acupressure though trained to perform it correctly might also be subjected to bias such as whether the procedure is performed on time.

## 5. Conclusions

Acupuncture combined with auricular acupressure could significantly increase the therapeutic effect of chloasma treatment and could be better than vitamins C and E. Combination treatment with auricular acupressure is worthy for more research to be carried out for future clinical practice of chloasma treatment.

## Figures and Tables

**Table 1:** Baseline characteristics.

Variable	Acupuncture + auricular acupressure (group A, *n* = 45)	Acupuncture (group B, *n* = 45)	Oral vitamin C and vitamin E (control) (group C, *n* = 45)	*P* value
*Age distribution, n (%)*				
18–29 yr	14 (31.1)	12 (26.7)	11 (24.4)	
30–39 yr	27 (60.0)	26 (57.8)	27 (60.0)	
40–49 yr	3 (6.7)	4 (8.9)	4 (8.9)	
50–59 yr	1 (2.2)	2 (4.4)	3 (6.7)	
≥60 yr	0 (0.0)	1 (2.2)	0 (0.0)	
Mean age (yr)	37.5 ± 5.2	38.1 ± 4.8	36.9 ± 5.0	0.11
*Weight (kg)*	56.2 ± 6.1	55.0 ± 5.9	56.1 ± 6.3	0.52
*Height (cm)*	159.1 ± 4.2	158.2 ± 4.1	158.1 ± 4.5	0.25
*Body mass index (kg/m* ^*2*^)	22.20 ± 2.0	21.97 ± 2.1	22.44 ± 2.2	0.31
*Duration of chloasma, n (%)*				
0–10 yr	35 (77.8)	33 (73.3)	36 (80.0)	
11–20 yr	10 (22.2)	11 (24.5)	9 (20.0)	
21–30 yr	0 (0.0)	1 (2.2)	0 (0.0)	
Mean duration (yr)	5.5 ± 1.2	6.0 ± 1.0	5.7 ± 1.3	0.21
*History of chloasma in a first-degree relative, n (%)*	19 (42.2)	21 (46.7)	20 (44.4)	0.18
*Marital status, n (%)*				*Total n (%)*
Single	11 (24.4)	16 (35.6)	13 (28.9)	40 (29.6)
Married	34 (75.6)	29 (64.4)	32 (71.1)	95 (70.4)
*Occupation, n (%)*				
Professional	25 (55.6)	24 (53.3)	26 (57.8)	75 (55.6%)
Housewife	20 (44.4)	21 (46.7)	19 (42.2)	60 (44.4%)
*Aggravating factors, n (%)*				
Hormonal therapy	1 (2.2)	0 (0.0)	1 (2.2)	2 (1.5)
Sun exposure	19 (42.2)	18 (40.0)	20 (44.4)	57 (42.2)
Cosmetic use	0 (0.0)	1 (2.2)	1 (2.2)	2 (1.5)
Smoking	0 (0.0)	0 (0.0)	0 (0.0)	0 (0.0)

*Note*. Data were presented as mean ± standard deviation or number of patients (*n*) with percentage (%).

**Table 2:** Evaluation of therapeutic effect.

	Acupuncture + auricular acupressure (group A, *n* = 45)	Acupuncture (group B, *n* = 45)	Oral vitamin C and vitamin E (control) (group C, *n* = 45)
*Area of skin lesion *			
Pretreatment score	2.3 ± 0.2	2.4 ± 0.2	2.3 ± 0.2
Posttreatment score	0.7 ± 0.15^*∗∗*††^	1.3 ± 0.15^*∗*†^	1.5 ± 0.1
*Color of skin lesion *			
Pretreatment score	2.0 ± 0.2	1.9 ± 0.2	2.1 ± 0.2
Posttreatment score	0.7 ± 0.15^*∗∗*††^	1.1 ± 0.15^*∗*†^	1.6 ± 0.1
*Treatment outcome, n (%)*			
Basically cured	34 (75.6)	28 (62.2)	17 (37.8)
Markedly effective	5 (11.1)	7 (15.6)	9 (20.0)
Effective	4 (8.9)	6 (13.3)	8 (17.8)
Ineffective	2 (4.4)	4 (8.9)	11 (24.4)
Total effective rate (%)	95.6^*∗∗*^	91.1^*∗*^	75.6

*Note*. Data were presented as mean ± standard deviation or number of patients (*n*) with percentage (%). Area and color of skin lesion: compared with the pretreatment level of the same group, ^*∗*^*P* < 0.05 and ^*∗∗*^*P* < 0.01; pre- and posttreatment difference compared with the control group; ^†^*P* < 0.05 and ^††^*P* < 0.01. Treatment outcome: compared with control group, ^*∗*^*P* < 0.05 and ^*∗∗*^*P* < 0.01.

**Table 3:** Hormone levels for pre- and posttreatment.

Group	Total cases (*n*)		FSH (mIU/mL)	LH (mIU/mL)	E_2_ (pg/mL)	P_4_ (ng/mL)
Acupuncture + auricular acupressure (group A)	45	Pretreatment	5.56 ± 1.08	4.32 ± 0.97	155 ± 5.60	8.85 ± 0.29
		Posttreatment	5.21 ± 0.98	4.58 ± 0.83	121 ± 5.25^*∗∗*††^	12.95 ± 0.28^*∗∗*††^
Acupuncture (group B)	45	Pretreatment	5.55 ± 1.18	4.32 ± 0.98	154 ± 5.65	8.88 ± 0.28
		Posttreatment	5.26 ± 1.21	4.50 ± 0.87	132 ± 5.32^*∗*†^	11.96 ± 0.27^*∗*†^
Oral vitamin C and vitamin E (control) (group C)	45	Pretreatment	5.51 ± 1.14	4.30 ± 0.96	156 ± 5.78	8.89 ± 0.31
		Posttreatment	5.31 ± 1.35	4.43 ± 0.88	140 ± 5.21	9.95 ± 0.29

*Note*. Data were presented as mean ± standard deviation, *n*: number of patients. Compared with the pretreatment level of the same group, ^*∗*^*P* < 0.05 and ^*∗∗*^*P* < 0.01; pre- and posttreatment difference compared with the control group, ^†^*P* < 0.05 and ^††^*P* < 0.01.
